# Clinic Reports

**Published:** 1880-07

**Authors:** L. D. Wilson


### CLINIC REPORTS.

*College of Medicine and Surgery, University of Michigan. Clinic of Professor McLean, February* 11*th. Reported by L. D. Wilson*

### TUMOR OF LOWER JAW.

Feb. 11, 1880, Mr. J. Reynolds, a healthy, strong man act. thirty-eight years, was presented with a large tumor, involving the entire right side of the lower jaw. It began in the submaxillary gland of that side, in May, 1879. It grew rapidly and soon involved the bone, the latter taking on the diseased condition in a remarkable degree. There had been but little pain up to October 1st. About that time pain set in with considerable severity and the tumor enlarging very rapidly. The molar teeth were removed several years ago. The teeth probably had nothing to do with the production of this tumor. There has been for about three months some discharge of pus from the surface at the base of the jaw, at a point opposite the location of the first molar. The mass extended about one and one-half inches below the natural base line of the jaw.

After thorough examination it was decided to remove the tumor, which would include the right half of the jaw. The patient was put under the influence of chloroform. The integuments covering the mass was dissected up so as to expose the whole outward aspect, then the jaw was divided at the symphasis, and the condyle dis articulated; then the whole mass, including the separated part of the jaw, was carefully dissected out and removed. A few small vessels were ligated, but very little blood was lost. The wound was dressed, the flaps that had been turned back were replaced; very little shock was experienced, and a rapid recovery was made. Ten days after the operation the external wound had entirely healed, and there was scarcely any disfigurement of the face.

